# Deep learning for pollen allergy surveillance from twitter in Australia

**DOI:** 10.1186/s12911-019-0921-x

**Published:** 2019-11-08

**Authors:** Jia Rong, Sandra Michalska, Sudha Subramani, Jiahua Du, Hua Wang

**Affiliations:** 1 Institute for Sustainable Industries & Liveable Cities, Victoria University, Ballarat Road, Melbourne, 3011 Australia; 2 Faculty of Information Technology, Monash University, Wellington Road, Melbourne, 3800 Australia

**Keywords:** Deep learning, Hay fever, Pollen allergy, Twitter mining

## Abstract

**Background:**

The paper introduces a deep learning-based approach for real-time detection and insights generation about one of the most prevalent chronic conditions in Australia - Pollen allergy. The popular social media platform is used for data collection as cost-effective and unobtrusive alternative for public health monitoring to complement the traditional survey-based approaches.

**Methods:**

The data was extracted from Twitter based on pre-defined keywords (i.e. ’hayfever’ OR ’hay fever’) throughout the period of 6 months, covering the high pollen season in Australia. The following deep learning architectures were adopted in the experiments: CNN, RNN, LSTM and GRU. Both default (GloVe) and domain-specific (HF) word embeddings were used in training the classifiers. Standard evaluation metrics (i.e. Accuracy, Precision and Recall) were calculated for the results validation. Finally, visual correlation with weather variables was performed.

**Results:**

The neural networks-based approach was able to correctly identify the implicit mentions of the symptoms and treatments, even unseen previously (accuracy up to 87.9% for GRU with GloVe embeddings of 300 dimensions).

**Conclusions:**

The system addresses the shortcomings of the conventional machine learning techniques with manual feature-engineering that prove limiting when exposed to a wide range of non-standard expressions relating to medical concepts. The case-study presented demonstrates an application of ’black-box’ approach to the real-world problem, along with its internal workings demonstration towards more transparent, interpretable and reproducible decision-making in health informatics domain.

## Background

### Introduction

According to Australian Institute of Health and Welfare (AIHW) [1], in 2014−15 nearly 1 in 5 Australian suffered from Pollen allergy, which amounts to 4.5 mln of citizens, predominantly working-aged adults. What is more, the expenditure on Allergic rhinitis medications doubled between 2001 and 2010, going from $107.8 mln to $226.8 mln per year, as reported by Australian pharmacies [1]. Overall allergies are increasing, but the reasons for an observed growth are not entirely clear [2, 3].

The potential of social media for public health mining has already been demonstrated in previous studies on Adverse Drug Reactions (ADRs) [4–8], antibiotics misuse [9], influenza detection [10–12], allergy surveillance [13–17], and so on. Still, the automatic approaches frequently under-perform when exposed to novel/creative phrases, sarcasm, ambiguity and misspellings [6, 18, 19]. Consequently, the conventional machine learning classifiers struggle with correct identification of non-medical expressions such as *’hay fever sob’* or *’dribbling nose’*, typical of social media discourse. On the other hand, the large proportion of user-generated content is of either commercial or informative nature - irrelevant for surveillance and knowledge discovery purposes. The news, warnings, products and services ads related to the condition can be published by both public as well as private accounts, limiting usability of the associated metadata. A critical challenge lies in abstracting essential information, in the context of Hay fever surveillance, from highly unstructured user-generated content to support public health monitoring from social media.

Deep learning emerged as a sub-field of machine learning and already benefited numerous Natural Language Processing (NLP) tasks [20]. The ability to learn the most salient aspects from text automatically eliminated the need for conventional classifiers dependent on manual feature-engineering. Further application of word embeddings allowed to account for syntactic and semantic regularities between the words, leading to classification performance improvement. As state-of-the-art approach, deep learning in public health mining domain is still in its infancy. Previous studies on allergies surveillance from social media conducted in the UK and US utilised either traditional machine learning classifiers such as Multinomial Naive Bayes [13, 17], or lexicon-based approaches [14–16]. The application of deep learning for Hay fever-related user-generated content identification and knowledge discovery about the condition in Australia is yet to be explored in the literature.

### Prevalence and severity of Hay fever

Pollen allergy, commonly known as Hay Fever, significantly reduces the quality of life and affects physical, psychological and social functioning. The symptoms experienced are caused by body’s immune response to the inhaled pollen, resulting in chronic inflammation of eyes and nasal passages. Nasal congestion is often associated with sleep disturbance, resulting in daytime fatigue and somnolence. An increased irritability and self-consciousness along with a decreased level of energy and alertness are frequently observed during pollen season [21]. Moderate and severe symptoms of Hay fever considerably impair learning ability in children, while adults suffer from work absences and reduced productivity [21, 22]. According to World Allergy Organisation (WAO) [22], Hay fever is increasing in prevalence and severity, and will continue to be a concern.

Around the world, in both developed and developing countries, environments are undergoing profound changes [3]. An increased air pollution and global warming have a substantial impact on respiratory health of the population. Ziska et al. [23] has already reported that the duration of ragweed pollen season has been increasing in recent decades in North America. Any potential pattern changes, including prolonged pollen season, increased intensity of allergens or un-expected pollens detection directly affect the physical, psychological and social functioning of allergy sufferers [22]. The response to the external factors further differs among the individuals, which is particularly exacerbated in countries with high migration rates [3]. As for 2015, approx. 30% of the Australia’s Estimated Resident Population (ERP) was born overseas [24].

The ever-changing and unpredictable nature of Pollen allergies evolution necessitates the accurate and timely statistics about the state of the condition. The conventional, survey-based approaches involve a fraction of the population, and incur significant reporting delays (approx. 1 year in the case of official government reports [1]). Alternative approaches involve the number of hospital admissions and General Practitioners (GPs) reports of Hay fever instances. According to the study conducted in New South Wales - Australia [25], ’patients believe that Allergic rhinitis is the condition that should be self-managed’. Bypassing the Health Care Professionals (HCPs) and reliance on over-the-counter drugs can lead to statistics derived from services under-estimation. Also, the pharmacies supply data of oral antihistamines - the common Hay fever medicine - is used to indicate yearly start and peak of the season [1, 2]. Despite insightful, such analyses are not conducted systematically as the collection of data from drug manufacturers/pharmacy outlets across the country is required. Finally, the pollen rates assist in estimations of starting and peaking points of allergy seasons. Still, the actual condition prevalence may vary due to different responses to particular allergens among individuals.

### Allergies surveillance from social media

Given the limitations of traditional approaches for allergies surveillance, the alternative sources of data increase in importance to closer reflect the state of the condition within the population. One domain that has grown by massive proportions in recent years, as well as continues to grow, is social media [6, 26]. Online platforms attract and encourage users to discuss their health issues, use of drugs, side effects and alternative treatments [6]. The updates range from generic signs of dissatisfaction (e.g. *’hay fever sucks’*) to specific symptoms description (e.g. *’my head is killing me’*). Also, it has been observed that individuals often prefer to share their health-related experiences with peers, rather than during clinical studies, or even physicians [27]. As a result, social media has become a source of valuable data, increasingly used for real-time detection and knowledge discovery [28].

Previous studies conducted in UK and US have already investigated the potential of Twitter for allergies surveillance. De Quincey et al. [15] observed that Twitter users are self-reporting the symptoms as well as medications, and the volume of Hay fever-related tweets strongly correlates (r=0.97, p<0.01) with incidents of Hay fever reported by Royal College of General Practitioners (RCGP) within the same year in the UK. Another correlation has been found in the work published by Cowie et al. [17], where the volume of Pollen allergy-related tweets collected in the UK over the period of 1 year resembled the pattern of pollen counts - grass pollen in particular. The study performed in the US has reported similar findings - strong correlations between (1) pollen rates and tweets reporting Hay fever symptoms (*r*=0.95), and (2) pollen rates and tweets reporting the use of antihistamines (*r*=0.93) [16]. Lee et al. [13] further observed the relationship between the weather conditions (daily maximum temperature), and number of conversations about allergies on Twitter. Additionally, the classification of actual allergy incidents and general awareness promotion was employed, along with the particular allergy types extraction. The correlations between the environmental factors and Hay fever-related tweets were also performed in the small-scale Australian study [29], where moderately strong dependencies were found for Temperature, Evaporation and Wind - all crucial factors in allergies development.

### Deep learning in text classification

Gao et al. [30] demonstrated how deep learning approach can improve model performance for multiple information extraction tasks from unstructured cancer pathology reports compared to conventional methods. The corpus of 2505 reports was manually annotated for (1) primary site (9 labels), and (2) histological grade (4 labels) identification. The models tested were RNN, CNN, LSTM and GRU, and word embeddings were implemented for word-to-vector representation. Another study explored the effectiveness of domain-specific word embeddings on classification performance in Adverse Drug Reactions (ADRs) extraction from social media [5]. The data was collected from Twitter and DailyStrength (the online support community dedicated to health issues), followed by annotation of total of 7663 posts for presence of (1) adverse reactions, (2) beneficial effects, (3) condition suffered, and (4) other symptoms. The use of word embeddings enabled even the non-medical expressions correct identification in highly informal social media streams. The improved performance following the domain-specific embeddings development was also demonstrated in the classification of ADRs-related [12] (medical embeddings), and crisis-related tweets [31] (crisis embeddings). The former employed the bi-directional LSTM model for detection of ADRs, Drug Entities and others. The latter used CNN model for binary identification of useful versus non-useful posts during a crisis event. Similarly, CNN was successfully applied in personality identification [32], sarcasm detection [33], aspect extraction [34] or emotion recognition [35].

CNNs capture the most salient n-gram information by means of its convolution and max-pooling operations. In terms of NLP tasks, RNNs are found particularly suitable due to the ability to process variable length inputs as well as long-distance word relationships [36]. In text classification, the dependencies between the center and far-away words can be meaningful and contribute towards performance improvement [37]. The LSTMs (Long Short-Term Memory), as variants of RNNs - can leverage both short and long-distance word relationships [37]. Unlike LSTMs, GRUs (Gated Recurrent Unit) fully expose their memory content each timestep, and whenever a previously detected feature, or the memory content is considered to be important for later use, the update gate will be closed to carry the current memory content across multiple timesteps [38]. Based on empirical results, GRUs outperformed LSTMs in terms of convergence in CPU time and in terms of parameter updates and generalisation by using fixed number of parameters for all models on selected datasets [39].

### Contributions

The main contributions of the study can be stated as follows:

- We introduce Deep Learning application in the context of Pollen Allergy surveillance from Social Media in place of currently dominant conventional Machine Learning classifiers;
- We focus on challenging informal vocabulary, which leads to condition under/over-estimation if unaddressed in place of the traditional limited keyword/lexicon-based approaches;
- We propose the fine-grained classification into 4 classes in place of the most common binary classifiers, i.e. Hay Fever-related/Hay Fever-non-related;
- We enrich the data with an extensive list of weather variables for potential patterns identification, where previous studies focus mainly on Temperature, and Pollen Rate.

## Methods

### Study design

The objectives of the study are as follows:

- Framework development for quantitative and qualitative Hay fever monitoring from Twitter;
- Evaluation of multiple deep learning architectures to online user-generated content classification;
- Domain-specific embeddings training and evaluation for accuracy performance improvement;
- Internal workings demonstration through the predictive probabilities and embeddings vectors investigation;
- Correlation with weather variables for patterns identification and future forecasting.

**Fig. 1:**
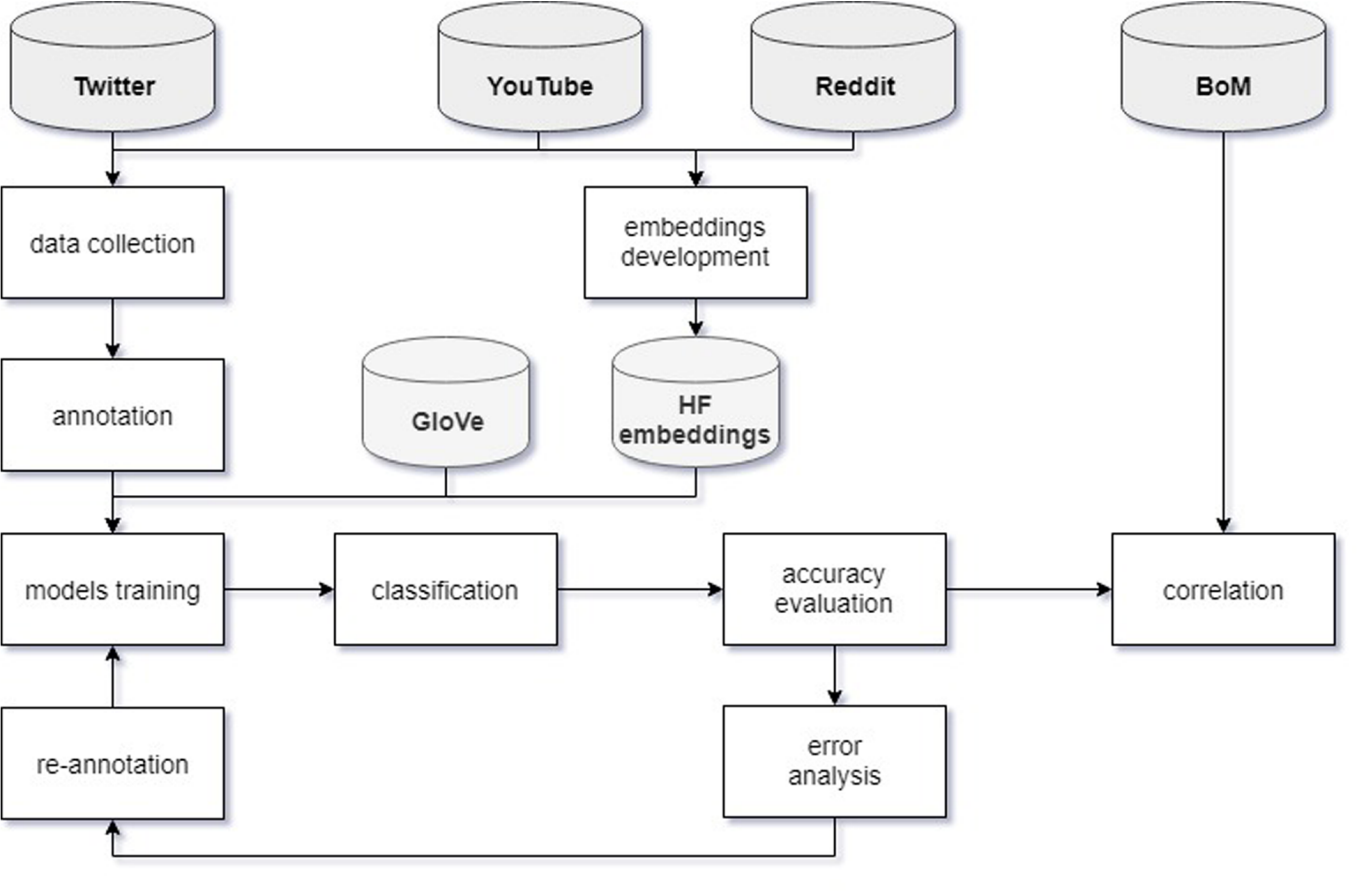
Methodology. Conceptual framework for data collection, tweets classification and weather correlation

The high-level methodological framework is presented in Fig. 1, and the particular steps are detailed in the following sub-sections.

### Data extraction

The extraction phase inlcuded the following stages:

#### Embeddings development

For the purpose of HF embeddings development, the relevant posts and comments from popular online platforms were crawled. The sources considered were: Twitter, YouTube and Reddit. In order to include only Hay fever-related data, the following keywords were searched for: *’hay fever’* OR *’hayfever’* OR *’pollen allergy’*. In the case of Twitter, the inclusion of pre-defined keywords in the content was required. As for YouTube and Reddit, the associated comments/posts from videos/threads that contained one or more keywords from the list in their titles were extracted. In total, approximately 22k posts were collected.

The following web crawling methods were applied based on the data sources used: (i) Twitter - TwitteR R package, (ii) Reddit - RedditExtractoR R package, and (iii) YouTube - NVivo. Gensim library for Python that provides access to Word2Vec training algorithms was used, with the window size set to 5. To enhance results reproducibility and inform future research, the details of the particular embeddings development schema implemented have been presented in Table 1.

**Table 1:** Embeddings development schema

	Twitter	Reddit	Youtube
Count	5,197	15,843	432
Average Character Length	145	209	88
St.Dev. Character Length	132	336	111
Minimum Date	2019/06/01	2009/12/02	2011/10/11
Maximum Date	2019/01/20	2019/01/20	2019/01/20

#### Target data

As the purpose of the study is Hay fever surveillance in Australia, the posts were extracted using the geo-coordinates of the following locations: (1) Alice Springs (radius=2,000mi), and (2) Sydney, Melbourne, and Brisbane (radius=300mi). Given that exact location extraction is practically unfeasible if geo-tag option was disabled, the separate datasets for (1) whole Australia, and (2) its major cities were created. The dataset 1 was used for classifier training, whereas dataset 2 was used for tweet volumes correlation with weather conditions for the particular area. Custom script was used to extract the data using R programming language and ‘TwitteR’ package. The posts were captured retrospectively at regular time intervals, and the parameters were as follows:

- Search terms: *’hayfever’* OR *’hay fever’*;
- Maximum number of tweets: *n*=1,000 (never reached due to limited number of posts meeting the specified criteria);
- Since/until dates: *s*=2018/06/01, *u*=2018/12/31 following the weekly schema;
- Geo-coordinates: Alice Springs (−23.698, 133.880), Sydney (−33.868, 151.209), Melbourne (−37.813, 144.963), and Brisbane (−27.469, 153.025).

The high precision was prioritised over the high recall, thus the very narrow scope of the search terms. After preliminary data exploration, wider list of search queries introduced an excessive noise to the dataset. For instance, the generic term ’allergy’ included other popular allergy types (i.e. Cats, Peanuts), and the specific symptoms such as ’sneezing’, ’runny nose’, ’watery eyes’ frequently referred to the other common conditions (i.e. Cold, Flu).

**Fig. 2:**
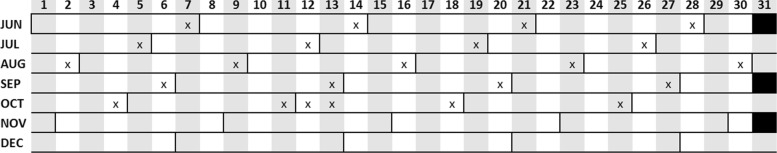
Data extraction calendar. Data collection period with ‘x’ indicating missing values

Data was obtained for 191 out of 214 days in total (89%). The posts from remaining 23 days were not captured due to technical issues^1^. Still, for quantitative analysis the missing values were accounted for to ensure findings validity. The compensation approach is detailed in sub-section Weather correlation, and the Extraction calendar is presented in Fig. 2, where ’x’ indicates the gaps in data collection. Qualitative analysis remained unaffected.

### Annotation process

The full dataset of 4,148 posts (Sydney - 1,040, Melbourne - 1928), and Brisbane - 222) was annotated by two researchers, active in health informatics domain. Annotators performed the evaluation using the tweet text as well as link to the online tweet version if text was unclear, where certain commonly occurring emojis provided further context for tweets interpretation, e.g. nose or tears. The approach followed the methodological considerations for undertaking Twitter research outlined by Colditz et al. [40]. In case of potential disagreements, either the consensus was obtained or the ‘Unrelated/Ambiguous’ class was selected. The inter-rater reliability was calculated using Cohen’s kappa statistic [41], taking into account the probability of agreement by chance. The score achieved was *κ* = 0.78 and is considered significant [42]. The usernames have been removed from the posts given the privacy considerations.

The study conducted by Lee et al. [13] categorised the allergy-related posts into the actual incidents of the condition and general awareness promotion. Analogically, the posts were annotated into Informative and Non-Informative, as detailed in Table 2. The Informative category split was introduced to allow for (1) personal detailed reporting, and (2) personal generic reporting separation. Class 1 was further used for symptoms and/or treatments extraction, whereas combined classes 1 and 2 were used for quantitative analysis of the condition prevalence estimation. The Non-Informative category included public broadcasting (3), and unrelated content (4).

**Table 2:** Annotation classes

Class	ID	Description	Example
Informative	1	Personal reporting including: symptoms (a), treatments (b) or both (c).	*‘90% sure we have either developed hayfever. My eyes feel so dry and my nose is running* whenever i go outside’ (a)
			*‘my hayfever better watch out today bc ate a spoonful of local honey AND took antihistamines’* (b)
			*‘Kings Cross triggered my hayfever today and now I can’t stop sneezing. I need a zyrtec’* (c)
	2	General personal reporting.	*‘Hayfever suuucks’*
Non-Informative	3	Public broadcasting including: marketing (a), news (b), warnings (c) etc.	*‘Himalayan Inhaler to help with asthma & hayfever #himalayansalt #onlineshopping’* (a)
			*‘Scientists in Melbourne have made a breakthrough that could change the lives of asthma and hay fever sufferers’* (b)
			*‘Be careful Victorians: thunderstorm asthma warning. Stay safe. #hayfever #health #bom #weather’* (c)
	4	Unrelated/Ambiguous.	*‘Very annoying. Could be a preservative or maybe hay fever.’*

### Training and testing

The experiments with 4 deep learning architectures were conducted due to various performances obtained on different datasets in previous studies. Pre-processing performed was minimal, and included removal of URLs, non-alphanumeric characters and lowercasing. In terms of emojis, their numerical representation was retained, following the punctuation removal. No excessive pre-processing was applied as models perform the operations on sequence of words in order they appear. Words are preserved in their original form without stemming/lemmatising due to their context-dependent representation, e.g. *’allergy’*, *’allergic’*, *’allergen’*. Also, Sarker et al. [6] suggested that stop words can play a positive effect on classifier performance. Analogical pre-processing steps were implemented for the embeddings development.

For feature extraction, the word-to-vector representation was adopted due to its ability to effectively capture the relationships between the words, thus proving superior in text classification tasks. Additionally, the use of word embeddings naturally extends the feature set, which is particularly advantageous in the case of small to moderate datasets. The 2 word embeddings variants were implemented (1) GloVe embeddings - as default, and (2) HF embeddings - as alternative. The pre-trained Common Crawl 840B tokens GloVe embeddings were downloaded from the website^2^. Both 50 dimensions (min) and 300 dimensions (max) options were tested. The HF embeddings were generated using 10 iterations and vector dimension of 50, given the moderate training data size. Previous study [4] reported improved classification performance with 50 dimensions while training domain-specific embeddings.

In terms of the parameters, the mini-batch size was set to default 32, the most popular non-linear activation function ReLU was selected, the number of recurrent units was set to standard 128, and the Nadam optimiser was used. The models were trained up to 50 epochs and implemented with open source neural network library Keras^3^.

Finally, the standard evaluation metrics were adopted, such as Accuracy, Precision (exactness) and Recall (completeness). The 5-fold cross-validation was followed, with 80:20 training and testing split as in [43]. The Confusion Matrices were further produced to examine in-detail the performances obtained for the particular classes.

### Weather correlation

As for the patterns investigation, the weather factors were superimposed on the tweet volume charts over the period of 6 months (2018/06/01−2018/12/31). The weekly averages of the number of Informative posts (class 1 + 2) were taken into account for Sydney, Melbourne, and Brisbane. The approach followed previous study conducted by Gesualdo et al. [16], where the weekly averages of tweets were used to avoid daily fluctuations for correlations with pollen rates and antihistamine prescriptions. The environmental data was obtained from Bureau of Meteorology^4^ (BOM) - Australia’s official weather forecast and weather radar. The following variables were extracted: Min Temp [ ^∘^ C], Max Temp [ ^∘^ C], Ave Temp [ ^∘^ C], Sunshine [hrs], Rainfall [mm], Evaporation [mm], Relative Humidity [%], Max Wind [km\h], Ave Wind [km\h] and Pressure (hPa). Analogically, the weekly averages were considered.

In the case of gaps in data collection (Fig. 2), the compensation approach was adopted, i.e. given 1 day-worth of data missing within the week, the average of the remaining 6 days was calculated and considered as the 7th day tweet volume. The weekly average was then estimated based on the complete 7-days record.

## Results

### Accuracy evaluation

The accuracies obtained for RNN, LSTM, CNN and GRU models are presented in Table 3. The default (GloVe) and alternative (HF) word embeddings options were considered. In terms of GloVe, the min (50) and max (300) number of dimensions were implemented. The highest accuracy was obtained for GRU model with GloVe embeddings of 300 dimensions (87.9%). Further evaluation metrics (Precision and Recall) were produced for GloVe/300 and HF/50 options, and are included in Table 4.

**Table 3:** Accuracy metrics

Embeddings	GloVe	GloVe	HF
Dimensions	50	300	50
RNN	0.659	0.637	0.677
LSTM	0.862	0.878	0.807
CNN	0.857	0.853	0.826
GRU	0.864	0.879	0.822

**Table 4:** Precision and Recall metrics

Embeddings/Dimensions	Model	Precision	Recall
GloVe/300	RNN	0.602	0.636
	LSTM	0.876	0.878
	CNN	0.854	0.850
	GRU	0.880	0.880
HF/50	RNN	0.688	0.676
	LSTM	0.804	0.806
	CNN	0.828	0.828
	GRU	0.824	0.822

### Classification output

The exemplary posts with the corresponding Classes, Classes ID, Predictive Probabilities and Post Implications are presented in Table 5. The implicit reference to either symptom or treatment is highlighted within each post. The official Hay fever symptoms list were extracted from Australasian Society of Clinical Immunology and Allergy (ASCIA) [21].

**Table 5:** Classification outputs

ID	Prob.	Example*	Implication**	Class
1	0.995	*I look like I just cried, but really it’s a hay fever*	watery eyes	Informative - symptom
1	0.588	*I’m getting hayfever sob no thank you*	watery eyes	Informative - symptom
1	0.993	*Tears and snot are streaming down my face at the moment #hayfever*	watery eyes, runny nose	Informative - symptom
1	0.996	*My hay fever is in overdrive today. Can’t stop sniffing*	runny nose	Informative - symptom
1	0.999	*@username I have an awful hay fever. Bad smells do not affect me during pollen season*	congested nose	Informative - symptom
1	0.503	*@username A really bad dose of hayfever that kept me up all last night. Not even herbal tea could help. Hopefully, it’ll ease down soon&hellip;*	sleep disturbance	Informative - symptom
1	0.994	*Thank hay fever in Melbourne today for me being awake at 4am. Existence is a real pain*	sleep disturbance	Informative - symptom
1	0.660	*me tries to sleep, me starts suffering from hayfever*	sleep disturbance	Informative - symptom
1	0.989	*i had hayfever for a week now and my lips have been getting dried up easilly so i have to lip it moist it*	new symptom	Informative - symptom
1	0.857	*Hayfever decided to turn more into cough and made me lose my voice a bit...*	new symptom	Informative - symptom
1	0.999	*Time to stock up on antihistamine* as hay fever season fast approaching	treatment (generic)	Informative - treatment
1	0.727	*@username Can’t wait for the spring, just have to remember to use my hay fever spray!*	treatment (generic)	Informative - treatment
1	0.999	*With Sudafed and plenty of water I have made it through the presention today! Hay fever won’t beat me anymore!*	treatment (specific)	Informative - treatment
1	0.999	*Too bad Loratadine is not helping with hay fever today.*	treatment (specific)	Informative - treatment
3	0.947	*EXCLUSIVE: @username shares his tips to alleviate watery eyes, an itchy or dribbling nose and constant coughing caused by hay fever.*	watery eyes, itchy nose	Non-Informative - news
3	0.999	*A study led by Prof Robyn O’Hehir has found a tablet used to treat chronic #hayfever could protect sufferers from future thunderstorm asthma events #immunotherapy*	treatment (generic)	Non-Informative - news
3	0.999	*Is TCM really better than Western medicine to help with hay fever? Chinese researchers think so. @username reports. #TCM #hayfever #medicine #Western #treatment*	treatment (generic)	Non-Informative - news
3	0.999	*A useful @username article speaking about the use of antihistamines in allergic rhinitis known as hay fever. it also inc. evidence of isotonic nasal salines that help to alleviate the antihistamine reliance, reduce symptoms and improve quality of life.*	treatment (generic)	Non-Informative - marketing
3	0.999	*New article about makeup tips for hayfever sufferers to conceal a red nose and itchy eyes has been posted on Daily Celeb Pics*	new symptom, itchy eyes	Non-Informative - marketing
4	0.821	*@username Tearing up or as Mr. Bart would say, "I’ve got a little bit of Hay fever".*	watery eyes	Non-Informative - unrelated
4	0.873	*I can sound like an echo of what has been written already: I’m really sorry you had to endure all of this, hope all the vacant passengers get nasty hayfever or even worse, also hope you get support if needed!*	generic	Non-Informative - ambiguous

* Usernames have been replaced with *@username* and posts have been obfuscated (paraphrased) due to privacy considerations.

** Symptoms defined according to Australasian Society of Clinical Immunology and Allergy (ASCIA), listed at https://www.allergy.org.au/patients/allergic-rhinitis-hay-fever-and-sinusitis/allergic-rhinitis-or-hay-fever.

Furthermore, the sample of outputs in the form of word-word co-occurrence statistics for both GloVe and HF embeddings were produced. Table 6 shows the top 15 terms with the highest associations with the following keywords: *’hayfever’*, *’antihistamines’* (as the most common Hay fever medication), *’eyes’* and *’nose’* (as the most affected body parts).

**Table 6:** Word embeddings

GloVe (300)								HF (50)			
’hayfever’		’antihistamines’		’eyes’		’nose’		’eyes’		’nose’	
rhinitis	0.725	antihistamine	0.836	eye	0.821	noses	0.704	nose	0.882	eyes	0.882
allergies	0.655	cetirizine	0.660	lips	0.741	nostrils	0.652	throat	0.853	throat	0.851
colds	0.627	sedating	0.657	smile	0.720	mouth	0.651	itchy	0.797	hair	0.792
allergy	0.617	medications	0.640	face	0.708	throat	0.639	sinuses	0.765	itchy	0.780
antihistamine	0.599	loratadine	0.627	ears	0.667	ears	0.638	sneezing	0.760	blocked	0.771
eczema	0.595	histamine	0.587	eyelids	0.650	lips	0.623	hair	0.756	dry	0.770
sneezing	0.595	rhinitis	0.581	hands	0.646	ear	0.613	hands	0.734	sinuses	0.764
allergic	0.587	medicines	0.579	staring	0.636	tongue	0.603	sore	0.725	face	0.758
sinusitis	0.586	stimulants	0.574	stared	0.632	nostril	0.594	watery	0.715	sneezing	0.742
asthma	0.583	antibiotics	0.572	tears	0.623	nasal	0.589	swollen	0.712	hands	0.737
pollens	0.581	histamines	0.571	eyed	0.619	eyes	0.576	blocked	0.712	mouth	0.727
bronchitis	0.568	fexofenadine	0.567	smiling	0.616	eye	0.565	dry	0.710	eye	0.705
antihistamines	0.559	hayfever	0.559	shining	0.600	lip	0.563	ears	0.702	head	0.699
excema	0.554	zyrtec	0.556	fingers	0.599	teeth	0.543	itching	0.690	ass	0.689
sniffles	0.552	pseudoephedrine	0.554	looked	0.597	head	0.540	runny	0.681	sore	0.675

### Error analysis

**Fig. 3:**
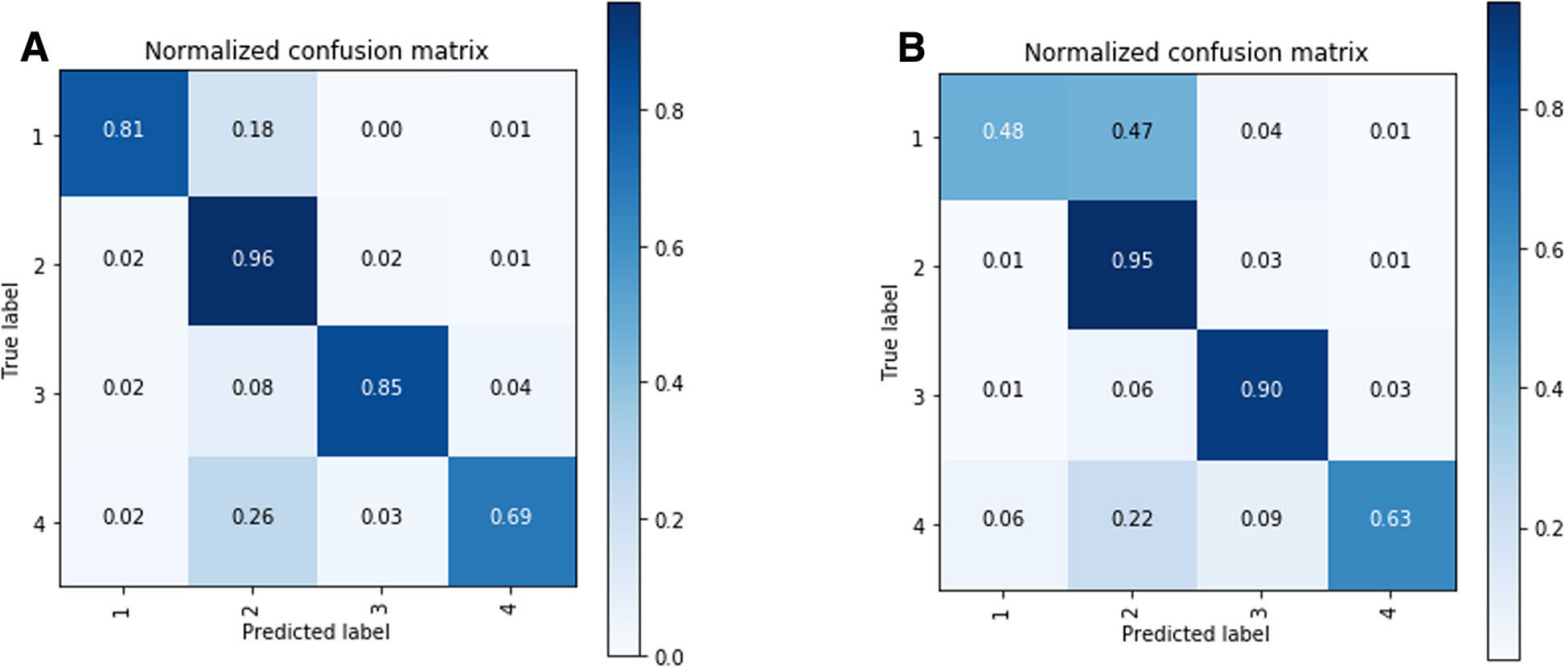
Confusion matrices. Normalised accuracy values among the respective classes. **a** GRU with GloVe Embeddings (300 Dimensions). **b** CNN with HF Embeddings (50 Dimensions)

In order to investigate the classification performance with respect to the particular classes, the confusion matrices were generated for both GloVe/300 and HF/50 options (Fig. 3). The highest performing deep learning architectures were selected according to the outputs presented in Table 4, i.e. GloVe/300 - GRU and HF/50 - CNN. Given different weights associated with the classes, the fine-grained performance examination facilitates the selection of the most suitable classifier based on the task at-hand. For instance, the performance achieved for classes 1 and 2 (Informative) is prioritised over the performance achieved for classes 3 and 4 (Non-Informative). The visual format of the analysis further assists the results interpretation.

In order to better understand the sources of misclassifications, the examples of inaccurate predictions were returned along with the corresponding classification probabilities (Table 7). The approach allows to obtain an insight behind the classifier confusion, and potentially re-annotate the falsely identified posts as part of the Active Learning towards classification performance improvement.

**Table 7:** Examples of misclassifications

No	Example	Prediction	Prob.	Actual
*P*_1_	*With this hay fever making me feel like I don’t have enough air in my lungs* I hope this helps;-)	2 Informative - generic	0.509	1 Informative - symptom
	Explanation: Rare expression of the symptom, referring to breathing difficulty, and negation ocurrence. Still, the low predictive probability obtained.			
P _2_	*How can I be awake with hay fever at 2am?!*	2 Informative - generic	0.543	1 Informative - symptom
	Explanation: Short length and expressiveness indicators (’?!’) typical for generic class 2. Still, the low predictive probability obtained.			
P _3_	*@username Same!!! Grass gives me bad rashes and hay fever as well as pollen and it sucksss*	2 Informative - generic	0.993	1 Informative - symptom
	Explanation: Repeated characters for added emphasis and expressiveness (’!!!’) typical for generic class 2. New and rare symptom occurred.			
P _4_	*Dubliner Goran Pavlovic claims started stinging himself with nettles and his hay fever symptoms started to disappear, if suffered would give it a try*	1 Informative - treatment	0.527	3 Non-Informative - news
	Explanation: Identified as treatment. The news content is paraphrased by the user, thus classified as personal. Still, the low predictive probability obtained.			
P _5_	*Could be a dangerous day tomorrow for asthmatic and hay fever sufferers. If we were in summer and not winter we would be looking at a severe fire danger. Those are warm winds.*	2 Informative - generic	0.863	3 Non-Informative - warning
	Explanation: Warning content paraphrased by the user - no typical ’warning’/’be careful’ phrase identified.			

### Weather correlation

**Fig. 4:**
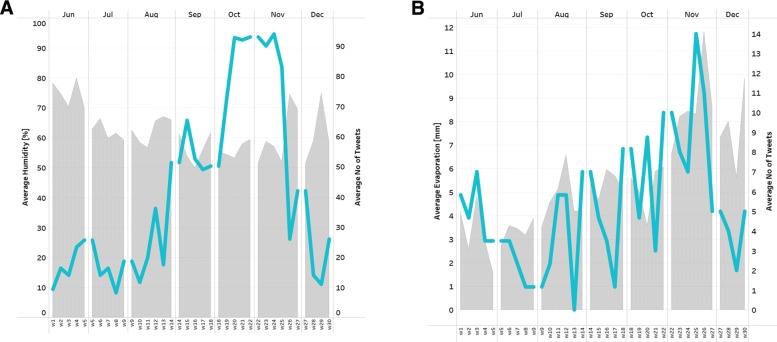
Visual correlation. The patterns between weather conditions (grey area) and volume of HF-related tweets (blue line). **a** Humidity [%] versus No of tweets in Melbourne. **b** Evaporation [mm] versus No of tweets in Brisbane

For potential patterns between environmental factors and HF-related Twitter activity, the graphs representing weekly averages of selected weather variables, and weekly averages of Informative tweets (class 1+2) throughout the 6 months period were produced. An interactive approach allowed to visually inspect the emerging correlations for Sydney, Melbourne and Brisbane. The most salient examples are presented in Fig. 4, where (a) the converse relationship between the Humidity [%] and volume of tweets, and (b) the relationship between the Evaporation [mm] and volume of tweets were observed. The Pearson’s correlation coefficients for the above mentioned examples were as follows (a) *r*=−0.24, *p*=0.009, and (b) *r*=0.22, *p*=0.027, both found statistically significant given the threshold of *p*<0.05 [see Additional file 1]. The normalisation procedure has been applied for calculating the inferential statistics. Also, the start as well as the peak of Hay fever season based on Twitter self-reports was indicated, e.g. Melbourne: beginning of September - start, October and November - peak.

## Discussion

### Deep learning approach validation

Deep learning approach has been adopted in order to account for the limitations of the lexicon-based and conventional machine learning techniques in accurate identification of non-standard expressions from social media, in the context of Hay fever. The maximum classification accuracy was achieved for GRU model with pre-trained GloVe embeddings of 300 dimensions (87.9%). The application of HF word embeddings did not improve the performance of the classifier, what can be attributed to relatively moderate training dataset size of (20k posts). Future work will investigate the large-scale domain-specific development, including data from online health communities (e.g. DailyStrength).

In the 1st part of the classification outputs (Table 5), the classifier was able to correctly identify the informal and often implicit references to syndromes (e.g. *’cried’*, *’tears’*, *’sniff’*, *’snot’*), and classify them as Informative - symptom (1). Only posts inclusive of *’hayfever’* OR *’hay fever’* keywords were considered to ensure they relevancy to the scope of the study. Additionally, the ’new’ symptoms (e.g. *’cough’*, *’lose my voice’*) have been recognised and classified as Informative - symptom (1). For consistency, the ’new’ have been defined as syndromes **not** occurring on the official website of Australasian Society of Clinical Immunology and Allergy [21]. Also, the medication-related terms ranging from generic in the level of granularity (*’spray’*, *’tablet’* etc.), to specific brand names (*’Sudafed’*, *’Zyrtec’* etc.) were recognised as treatments, proving the flexibility of the approach. Despite correct classification, the lower predictive probabilities were obtained for very rare expressions such as *’hay fever sob’* - 0.588 (watery eyes) or *’kept me up all night’* 0.503 (sleep disturbance).

In the 2nd part of the classification outputs (Table 5), the examples of accurately classified posts despite the confusing content implication are presented. For instance, the advertisement post including distinct Hay fever symptoms such as *’red nose’* and *’itchy eyes’* was classified correctly as Non-Informative - marketing (3), preventing it from further analysis and condition prevalence over-estimation.

With relatively small training dataset (approx. 4,000), the model proves its robustness in capturing the subtle regularities within the dataset. Lack of reliance on the external, pre-defined lexicons makes it suitable for emerging symptoms and treatments detection. Deep learning eliminates manual feature engineering effort, facilitating more automated and systematic approach. The ability to produce text representation selective to the aspects important for discrimination, but invariant to irrelevant factors is essential given highly noisy character of social media data. The traditional approaches, commonly referred to as ’shallow processing’, allow only for surface-level feature extraction, which proves effective for well-structured documents, but frequently fails when exposed to more challenging user-generated content. Thus, the advanced techniques are required if the minor and often latentdetails are decisive of the correct class assignment.

In order to obtain greater insight into the classification process, the word embeddings outputs were produced for the following keywords *’hayfever’*, *’antihistamines’*, *’eyes’* and *’nose’* (Table 6). In terms of the *’hayfever’*, mostly synonyms (e.g. *’rhinitis’*), plurals (e.g. *’allergies’*) or derivatives (e.g. *’allergic’*) were captured, accounting for their inter-dependance. The general term *’antihistamines’* demonstrated close relationship with specific Hay fever drugs (e.g. *’Cetirizine’, ’Loratadine’, ’Zyrtec’*), proving effective in identification of treatments non-identified a priori. The equivalent expressions such as *’eyelids’*, *’nostril’* have been found associated with the most commonly affected by Pollen allergy body parts, i.e. eyes and nose. Despite the linguistic variety abound on social media, the deep learning-based system with word embeddings demonstrated its ability to recognise the linkages between the concepts, essential for any NLP task.

On the other hand, the HF embeddings returned mostly symptoms related to particular organs (e.g. itchy, watery, blocked etc.), which can be considered informative for syndromic surveillance. Still, due to numerous symptoms occurring at once in the extracted posts, it is difficult to distinguish which body part does the particular symptom relates to. Furthermore, the embeddings outputs analysis can be found beneficial for informal health-related expressions mining. As stated by Velardi et al. [44], the knowledge of symptoms experienced is equally important as the language used to describe them. Finally, the model trained on causal language prevalent on social media faciltates more robust symptom-driven, rather than disease-driven surveillance approaches [44].

For continuous performance improvement, the concept of Active Learning was incorporated. The misclassified posts are returned along with the corresponding predictive probabilities, allowing for sources of classifier confusion identification and potential classes refinement. The sample of incorrectly identified posts with brief explanation is presented in Table 7.

### Knowledge discovery about Hay fever

Deep learning-based classification allows to effectively and efficiently extract the relevant information from large volume of streaming data. The real-time analysis is crucial for disease surveillance purposes. After posts classification into Informative and Non-Informative groups, the prevalence can be accurately estimated following the discard of news, advertisements, or ambiguous content. The finer-grained identification of (1) detailed symptoms/treatments versus (2) generic Hay fever mentions enables further knowledge discovery about the condition severity from the relevant class (1). The combined classes 1 and 2 allow for the quantitative prevalence estimation. As an example, the volume of HF-related tweets in Melbourne peaked in October and November, paralleling the findings obtained by the Australian Institute for Health and Welfare [1] regarding the wholesale supply of antihistamines sold throughout the year. The results prove useful for seasonality in pollen season estimation, accounting for its unpredictable and ever-changing pattern.

As for the correlation with weather factors, the converse relationship has been observed between Humidity [%] and Hay Fever self-reports in Melbourne. Also, the close dependency has been found in Brisbane, where volume of HF-related posts approximated the pattern of Evaporation variable [mm]. It can be attributed to the fact that plants are most likely to release the pollen into the air more on a sunny, rather than rainy day [29]. Thus, the proof-of-concept for future forecasting model was demonstrated.

## Conclusions

The state-of-the-art Deep Learning approach has been applied and validated in the context of Australian Hay fever surveillance from Twitter, following its superior performance on text classification tasks over conventional machine learning techniques. The rationale behind social media as a data source is based on the assumption that real-time events are reflected immediately on such platforms [12], showing advantage over time and cost-consuming survey-based approaches. The Pollen Allergy Surveillance System (PASS) has been introduced to further address the challenges of lexicon-based methods, reliant on pre-defined dictionaries and limited in their ability of emerging symptoms/treatments detection. Deep learning-based approach with word embeddings has allowed to capture both syntactic (e.g. *’allergy’*, *’allergen’*) and semantic (e.g. *’pollen allergy’*, *’allergic rhinitis’*) associations between the words, thus proving effective on highly unstructured social media streams. The implicit references to symptoms and treatments as well as non-medical expressions have been correctly identified (accuracy of up to 87.9%). Also, the irrelevant Hay fever-related content such as news or advertisement has been recognised as Non-Informative.

Overall, the framework consisting of (i) quantitative analysis (volume of relevant posts per time/space for prevalence estimation), and (ii) qualitative analysis (text mining-based severity evaluation) has been presented. The in-depth investigation of predictive probabilities and embeddings weights on the real-world example has provided an insight into the internal workings of the classifier. For instance, the top similar terms associated with HF-related keywords were produced to demonstrate *why* the selected approach worked, i.e. the vector for ’antihistamines’ included a wide range of specific medications’ brands, proving suitable for the *emerging* treatments discovery - valuable information for the robust Pollen Allergy Surveillance System development. Finally, the system has allowed to minimise the risk of Hay fever under/over-estimation, while incorporating increasingly popular social media data for public health exploration purposes.

## Supplementary information

**Additional file 1** Pearson’s coefficients for correlation with weather variables.

## Data Availability

The dataset used in this study is available from the corresponding author upon reasonable request.

## Abbreviations

ADR: Adverse drug reactions
AIHW: Australian institute of health and welfare
AR: Allergic rhinitis
ASCIA: Australasian society of clinical immunology and allergy
CNN: Convolutional neural network
DL: Deep learning
ERP: Estimated resident population
GloVe: Global vectors for word representation
GRU: Gated recurrent unit
HCP: Health-care professional
HF: Hay fever
LSTM: Long-short term memory
ML: Machine learning
NLP: Natural language processing
RNN: Recurrent neural network
WHO: World Health Organization

## Footnotes

1 ’Until date’ parameter excludes tweets posted on that date, of which the authors have been unaware (therefore the end-of-week gaps). It has been realised and corrected from October onwards.

2
https://nlp.stanford.edu/projects/glove/

3
https://keras.io/

4
http://www.bom.gov.au/

## References

[1] Australian Institute of Health and Welfare (AIHW). Allergic rhinitis (’hay fever’). 2016. https://www.aihw.gov.au/reports/chronic-respiratory-conditions/allergic-r%hinitis-hay-fever/contents/ allergic-rhinitis-by-the-numbers. Accessed 30 Jan 2019.

[2] Vigo M, Hassan L, Vance W, Jay C, Brass A, Cruickshank S (2017). Britain breathing: using the experience sampling method to collect the seasonal allergy symptoms of a country. J Am Med Informa Assoc.

[3] D’Amato G, Holgate ST, Pawankar R, Ledford DK, Cecchi L, Al-Ahmad M, Al-Enezi F, Al-Muhsen S, Ansotegui I, Baena-Cagnani CE (2015). Meteorological conditions, climate change, new emerging factors, and asthma and related allergic disorders. a statement of the world allergy organization. World Allergy Org J.

[4] Xia L, Wang GA, Fan W (2017). A deep learning based named entity recognition approach for adverse drug events identification and extraction in health social media. International Conference on Smart Health.

[5] Nikfarjam A, Sarker A, O’connor K, Ginn R, Gonzalez G (2015). Pharmacovigilance from social media: mining adverse drug reaction mentions using sequence labeling with word embedding cluster features. J Am Med Informa Assoc.

[6] Sarker A, Gonzalez G (2015). Portable automatic text classification for adverse drug reaction detection via multi-corpus training. J Biomed Informa.

[7] Patki A, Sarker A, Pimpalkhute P, Nikfarjam A, Ginn R, O’Connor K, Smith K, Gonzalez G (2014). Mining adverse drug reaction signals from social media: going beyond extraction. Proc BioLinkSig.

[8] Jonnagaddala J, Jue TR, Dai H-J (2016). Binary classification of twitter posts for adverse drug reactions. Proceedings of the Social Media Mining Shared Task Workshop at the Pacific Symposium on Biocomputing, Big Island, HI, USA.

[9] Scanfeld D, Scanfeld V, Larson EL (2010). Dissemination of health information through social networks: Twitter and antibiotics. Am J Infect Cont.

[10] Byrd K, Mansurov A, Baysal O (2016). Mining twitter data for influenza detection and surveillance. Proceedings of the International Workshop on Software Engineering in Healthcare Systems.

[11] Culotta A (2010). Towards detecting influenza epidemics by analyzing twitter messages. Proceedings of the First Workshop on Social Media Analytics.

[12] Wang C-K, Singh O, Tang Z-L, Dai H-J (2017). Using a recurrent neural network model for classification of tweets conveyed influenza-related information. Proceedings of the International Workshop on Digital Disease Detection Using Social Media 2017 (DDDSM-2017).

[13] Lee K, Agrawal A, Choudhary A (2015). Mining social media streams to improve public health allergy surveillance. 2015 IEEE/ACM International Conference on Advances in Social Networks Analysis and Mining (ASONAM).

[14] de Quincey E (2014). Potential of social media to determine hay fever seasons and drug efficacy. Planet@ Risk.

[15] de Quincey E, Kyriacou T, Pantin T (2016). # hayfever; a longitudinal study into hay fever related tweets in the uk. Proceedings of the 6th International Conference on Digital Health Conference.

[16] Gesualdo F, Stilo G, D’Ambrosio A, Carloni E, Pandolfi E, Velardi P, Fiocchi A, Tozzi AE (2015). Can twitter be a source of information on allergy? correlation of pollen counts with tweets reporting symptoms of allergic rhinoconjunctivitis and names of antihistamine drugs. PloS One.

[17] Cowie S, Arthur R, Williams H (2018). @ choo: Tracking pollen and hayfever in the uk using social media. Sensors.

[18] Leaman R, Wojtulewicz L, Sullivan R, Skariah A, Yang J, Gonzalez G (2010). Towards internet-age pharmacovigilance: extracting adverse drug reactions from user posts to health-related social networks. Proceedings of the 2010 Workshop on Biomedical Natural Language Processing.

[19] Edwards IR, Lindquist M (2011). Social media and networks in pharmacovigilance. Drug Saf.

[20] Collobert R, Weston J, Bottou L, Karlen M, Kavukcuoglu K, Kuksa P (2011). Natural language processing (almost) from scratch. J Mach Learn Res.

[21] Australasian Society of Clinical Immunology and Allergy (ASCIA). Pollen allergy. 2017. https://www.allergy.org.au/images/pcc/ASCIA_PCC_Pollen_allergy_2017.pdf. Accessed: 2019 Jan 30.

[22] World Allergy Organization (WAO). World Allergy Week 2016. 2016. https://www.worldallergy.org/UserFiles/file/WorldAllergyWeek2016FactSheet%.pdf. Accessed: 2019 Jan 30.

[23] Ziska L, Knowlton K, Rogers C, Dalan D, Tierney N, Elder MA, Filley W, Shropshire J, Ford LB, Hedberg C (2011). Recent warming by latitude associated with increased length of ragweed pollen season in central north america. Proc Nat Acad Sci.

[24] Australian Bureau of Statistics (ABS). Migration, Australia, 2014-15. 2016. http://www.abs.gov.au/AUSSTATS/abs@.nsf/Lookup/3412.0Main+Features12014-1%5?OpenDocument. Accessed: 2019 Jan 30.

[25] Cvetkovski B, Kritikos V, Yan K, Bosnic-Anticevich S (2018). Tell me about your hay fever: a qualitative investigation of allergic rhinitis management from the perspective of the patient. NPJ Primary Care Respiratory Med.

[26] Ginn R, Pimpalkhute P, Nikfarjam A, Patki A, O’Connor K, Sarker A, Smith K, Gonzalez G. Mining twitter for adverse drug reaction mentions: a corpus and classification benchmark. In: Proceedings of the Fourth Workshop on Building and Evaluating Resources for Health and Biomedical Text Processing. Citeseer: 2014.

[27] Davison KP, Pennebaker JW, Dickerson SS (2000). Who talks?the social psychology of illness support groups. Am Psych.

[28] Tuarob S, Tucker CS, Salathe M, Ram N (2014). An ensemble heterogeneous classification methodology for discovering health-related knowledge in social media messages. J Biomed Informa.

[29] Subramani S, Michalska S, Wang H, Whittaker F, Heyward B (2018). Text mining and real-time analytics of twitter data: A case study of australian hay fever prediction. International Conference on Health Information Science.

[30] Gao S, Young MT, Qiu JX, Yoon H-J, Christian JB, Fearn PA, Tourassi GD, Ramanthan A (2017). Hierarchical attention networks for information extraction from cancer pathology reports. J Am Med Informa Assoc.

[31] Nguyen DT, Al Mannai KA, Joty S, Sajjad H, Imran M, Mitra P (2017). Robust classification of crisis-related data on social networks using convolutional neural networks. Eleventh International AAAI Conference on Web and Social Media.

[32] Majumder N, Poria S, Gelbukh A, Cambria E (2017). Deep learning-based document modeling for personality detection from text. IEEE Intell Syst.

[33] Poria S, Cambria E, Hazarika D, Vij P. A deeper look into sarcastic tweets using deep convolutional neural networks. arXiv preprint arXiv:1610.08815. 2016.

[34] Poria S, Cambria E, Gelbukh A (2016). Aspect extraction for opinion mining with a deep convolutional neural network. Knowl-Based Syst.

[35] Poria S, Chaturvedi I, Cambria E, Hussain A (2016). Convolutional mkl based multimodal emotion recognition and sentiment analysis. 2016 IEEE 16th International Conference on Data Mining (ICDM).

[36] Goller C, Kuchler A (1996). Learning task-dependent distributed representations by backpropagation through structure. Proceedings of International Conference on Neural Networks (ICNN’96), vol 1.

[37] Gers FA, Schmidhuber J, Cummins F (1999). Learning to forget: Continual prediction with lstm. 9th International Conference on Artificial Neural Networks: ICANN ’99.

[38] Cho K, Van Merriënboer B, Gulcehre C, Bahdanau D, Bougares F, Schwenk H, Bengio Y. Learning phrase representations using rnn encoder-decoder for statistical machine translation. arXiv preprint arXiv:1406.1078. 2014.

[39] Chung J, Gulcehre C, Cho K, Bengio Y. Empirical evaluation of gated recurrent neural networks on sequence modeling. CoRR. 2014; abs/1412.3555. http://arxiv.org/abs/1412.3555. https://dblp.org/rec/bib/journals/corr/ChungGCB14.

[40] Colditz JB, Chu K-H, Emery SL, Larkin CR, James AE, Welling J, Primack BA (2018). Toward real-time infoveillance of twitter health messages. Am J Publ Health.

[41] Carletta J (1996). Assessing agreement on classification tasks: the kappa statistic. Comput Linguistics.

[42] Viera AJ, Garrett JM (2005). Understanding interobserver agreement: the kappa statistic. Fam Med.

[43] Serban O, Thapen N, Maginnis B, Hankin C, Foot V (2019). Real-time processing of social media with sentinel: a syndromic surveillance system incorporating deep learning for health classification. Inf Process Manag.

[44] Velardi P, Stilo G, Tozzi AE, Gesualdo F (2014). Twitter mining for fine-grained syndromic surveillance. Artif Intell Med.

