# Supplementary material for: Deep learning for pollen allergy surveillance from twitter in Australia
Source: BMC Med Inform Decis Mak. 2019 Nov 8;19:208. doi: 10.1186/s12911-019-0921-x (PMC6839169; doi:10.1186/s12911-019-0921-x)
Supplement: Supplementary file 1 — Additional file 1 Pearson’s coefficients for correlation with weather variables. [file 12911_2019_921_MOESM1_ESM.docx]

| SUMMARY OUTPUT **(complete cases for Melbourne, Sydney and Brisbane (605 out of 642))** | |  |  |  |  |  |  |  |
| --- | --- | --- | --- | --- | --- | --- | --- | --- |
|  |  |  |  |  |  |  |  |  |
| *Regression Statistics* | |  |  |  |  |  |  |  |
| Multiple R | 0.473594 |  |  |  |  |  |  |  |
| R Square | 0.224291 |  |  |  |  |  |  |  |
| Adjusted R Square | 0.207865 |  |  |  |  |  |  |  |
| Standard Error | 0.888861 |  |  |  |  |  |  |  |
| Observations | 605 |  |  |  |  |  |  |  |
|  |  |  |  |  |  |  |  |  |
| ANOVA |  |  |  |  |  |  |  |  |
|  | *df* | *SS* | *MS* | *F* | *Significance F* |  |  |  |
| Regression | 12 | 135.696 | 11.308 | 17.17509 | 9.52E-32 |  |  |  |
| Residual | 594 | 469.304 | 0.790074 |  |  |  |  |  |
| Total | 606 | 605 |  |  |  |  |  |  |
|  |  |  |  |  |  |  |  |  |
|  | *Coefficients* | *Standard Error* | *t Stat* | *P-value* | *Lower 95%* | *Upper 95%* | *Lower 95.0%* | *Upper 95.0%* |
| Intercept | -1.3E-15 | 0.036137 | -3.5E-14 | 1 | -0.07097 | 0.070973 | -0.07097 | 0.070973 |
| Rainfall (mm) | 0.00378 | 0.039301 | 0.09618 | 0.92341 | -0.07341 | 0.080966 | -0.07341 | 0.080966 |
| Evaporation (mm) | 0.221091 | 0.05205 | 4.247646 | 2.51E-05 | 0.118866 | 0.323316 | 0.118866 | 0.323316 |
| Sunshine (hours) | 0.296449 | 0.063803 | 4.646332 | 4.16E-06 | 0.171143 | 0.421756 | 0.171143 | 0.421756 |
| Speed of max wind (km/h) | 0.149501 | 0.067873 | 2.202671 | 0.028001 | 0.016202 | 0.282801 | 0.016202 | 0.282801 |
| ave temp | 0 | 0 | 65535 | - | 0 | 0 | 0 | 0 |
| sd temp | 0 | 0 | 65535 | - | 0 | 0 | 0 | 0 |
| min temp | -0.47491 | 0.065108 | -7.29425 | - | -0.60278 | -0.34704 | -0.60278 | -0.34704 |
| max temp | 0.04177 | 0.062836 | 0.664744 | 0.506472 | -0.08164 | 0.165178 | -0.08164 | 0.165178 |
| ave humidity | 0.141353 | 0.057629 | 2.452822 | 0.01446 | 0.028172 | 0.254534 | 0.028172 | 0.254534 |
| ave wind | 0.096249 | 0.062918 | 1.529742 | 0.126613 | -0.02732 | 0.219818 | -0.02732 | 0.219818 |
| ave cloud | 0.520976 | 0.062819 | 8.293294 | 7.39E-16 | 0.397601 | 0.64435 | 0.397601 | 0.64435 |
| ave pressure | 0.095124 | 0.047273 | 2.012218 | 0.044648 | 0.002281 | 0.187967 | 0.002281 | 0.187967 |

| SUMMARY OUTPUT **(complete cases for Melbourne (203 out of 214))** | |  |  |  |  |  |  |  |
| --- | --- | --- | --- | --- | --- | --- | --- | --- |
|  |  |  |  |  |  |  |  |  |
| *Regression Statistics* | |  |  |  |  |  |  |  |
| Multiple R | 0.595756 |  |  |  |  |  |  |  |
| R Square | 0.354925 |  |  |  |  |  |  |  |
| Adjusted R Square | 0.310911 |  |  |  |  |  |  |  |
| Standard Error | 0.825853 |  |  |  |  |  |  |  |
| Observations | 203 |  |  |  |  |  |  |  |
|  |  |  |  |  |  |  |  |  |
| ANOVA |  |  |  |  |  |  |  |  |
|  | *df* | *SS* | *MS* | *F* | *Significance F* |  |  |  |
| Regression | 12 | 72.04975 | 6.004146 | 10.56397 | 6.82E-16 |  |  |  |
| Residual | 192 | 130.9503 | 0.682033 |  |  |  |  |  |
| Total | 204 | 203 |  |  |  |  |  |  |
|  |  |  |  |  |  |  |  |  |
|  | *Coefficients* | *Standard Error* | *t Stat* | *P-value* | *Lower 95%* | *Upper 95%* | *Lower 95.0%* | *Upper 95.0%* |
| Intercept | 8.04E-15 | 0.057963 | 1.39E-13 | 1 | -0.11433 | 0.114327 | -0.11433 | 0.114327 |
| Rainfall (mm) | 0.043569 | 0.064405 | 0.676494 | 0.499541 | -0.08346 | 0.170601 | -0.08346 | 0.170601 |
| Evaporation (mm) | 0.281536 | 0.087349 | 3.223125 | 0.00149 | 0.10925 | 0.453823 | 0.10925 | 0.453823 |
| Sunshine (hours) | 0.358508 | 0.099332 | 3.609175 | 0.000392 | 0.162585 | 0.554431 | 0.162585 | 0.554431 |
| Speed of max wind (km/h) | -0.04402 | 0.119513 | -0.36829 | 0.713063 | -0.27974 | 0.191712 | -0.27974 | 0.191712 |
| ave temp | 0 | 0 | 65535 | - | 0 | 0 | 0 | 0 |
| sd temp | 0 | 0 | 65535 | - | 0 | 0 | 0 | 0 |
| min temp | -0.10817 | 0.092263 | -1.17244 | - | -0.29015 | 0.073806 | -0.29015 | 0.073806 |
| max temp | 0.142178 | 0.087077 | 1.63279 | 0.104152 | -0.02957 | 0.313928 | -0.02957 | 0.313928 |
| ave humidity | -0.24266 | 0.091262 | -2.65892 | 0.008501 | -0.42266 | -0.06265 | -0.42266 | -0.06265 |
| ave wind | -0.14036 | 0.102499 | -1.3694 | 0.172474 | -0.34253 | 0.061806 | -0.34253 | 0.061806 |
| ave cloud | 0.413171 | 0.092709 | 4.456656 | 1.41E-05 | 0.230313 | 0.596029 | 0.230313 | 0.596029 |
| ave pressure | 0.102903 | 0.085331 | 1.205918 | 0.229333 | -0.0654 | 0.27121 | -0.0654 | 0.27121 |

| SUMMARY OUTPUT **(complete cases for Brisbane (207 out of 214))** | |  |  |  |  |  |  |  |
| --- | --- | --- | --- | --- | --- | --- | --- | --- |
|  |  |  |  |  |  |  |  |  |
| *Regression Statistics* | |  |  |  |  |  |  |  |
| Multiple R | 0.472287 |  |  |  |  |  |  |  |
| R Square | 0.223055 |  |  |  |  |  |  |  |
| Adjusted R Square | 0.173211 |  |  |  |  |  |  |  |
| Standard Error | 0.905842 |  |  |  |  |  |  |  |
| Observations | 207 |  |  |  |  |  |  |  |
|  |  |  |  |  |  |  |  |  |
| ANOVA |  |  |  |  |  |  |  |  |
|  | *df* | *SS* | *MS* | *F* | *Significance F* |  |  |  |
| Regression | 12 | 46.17234 | 3.847695 | 5.627003 | 2.92E-08 |  |  |  |
| Residual | 196 | 160.8277 | 0.820549 |  |  |  |  |  |
| Total | 208 | 207 |  |  |  |  |  |  |
|  |  |  |  |  |  |  |  |  |
|  | *Coefficients* | *Standard Error* | *t Stat* | *P-value* | *Lower 95%* | *Upper 95%* | *Lower 95.0%* | *Upper 95.0%* |
| Intercept | -6.3E-16 | 0.06296 | -9.9E-15 | 1 | -0.12417 | 0.124167 | -0.12417 | 0.124167 |
| Rainfall (mm) | 0.083149 | 0.074566 | 1.115104 | 0.266171 | -0.06391 | 0.230204 | -0.06391 | 0.230204 |
| Evaporation (mm) | 0.217728 | 0.097704 | 2.228455 | 0.026987 | 0.025043 | 0.410414 | 0.025043 | 0.410414 |
| Sunshine (hours) | 0.214605 | 0.135266 | 1.586539 | 0.114229 | -0.05216 | 0.481369 | -0.05216 | 0.481369 |
| Speed of max wind (km/h) | 0.238935 | 0.102924 | 2.321481 | 0.021289 | 0.035955 | 0.441915 | 0.035955 | 0.441915 |
| ave temp | 0 | 0 | 65535 | - | 0 | 0 | 0 | 0 |
| sd temp | 0.064419 | 0.118687 | 0.542763 | - | -0.16965 | 0.298486 | -0.16965 | 0.298486 |
| min temp | 0 | 0 | 65535 | - | 0 | 0 | 0 | 0 |
| max temp | -0.0707 | 0.134971 | -0.5238 | - | -0.33688 | 0.195484 | -0.33688 | 0.195484 |
| ave humidity | -0.02104 | 0.118601 | -0.17744 | 0.859348 | -0.25494 | 0.212853 | -0.25494 | 0.212853 |
| ave wind | -0.03681 | 0.0998 | -0.36879 | 0.712679 | -0.23363 | 0.160014 | -0.23363 | 0.160014 |
| ave cloud | 0.321964 | 0.129729 | 2.481826 | 0.013912 | 0.066121 | 0.577808 | 0.066121 | 0.577808 |
| ave pressure | -0.0819 | 0.107587 | -0.7612 | 0.447451 | -0.29407 | 0.130281 | -0.29407 | 0.130281 |

| SUMMARY OUTPUT **(complete cases for Sydney (195 out of 214))** | |  |  |  |  |  |  |  |
| --- | --- | --- | --- | --- | --- | --- | --- | --- |
|  |  |  |  |  |  |  |  |  |
| *Regression Statistics* | |  |  |  |  |  |  |  |
| Multiple R | 0.451166 |  |  |  |  |  |  |  |
| R Square | 0.203551 |  |  |  |  |  |  |  |
| Adjusted R Square | 0.149396 |  |  |  |  |  |  |  |
| Standard Error | 0.918729 |  |  |  |  |  |  |  |
| Observations | 195 |  |  |  |  |  |  |  |
|  |  |  |  |  |  |  |  |  |
| ANOVA |  |  |  |  |  |  |  |  |
|  | *df* | *SS* | *MS* | *F* | *Significance F* |  |  |  |
| Regression | 12 | 39.69242 | 3.307701 | 4.702542 | 1.16E-06 |  |  |  |
| Residual | 184 | 155.3076 | 0.844063 |  |  |  |  |  |
| Total | 196 | 195 |  |  |  |  |  |  |
|  |  |  |  |  |  |  |  |  |
|  | *Coefficients* | *Standard Error* | *t Stat* | *P-value* | *Lower 95%* | *Upper 95%* | *Lower 95.0%* | *Upper 95.0%* |
| Intercept | 2.06E-14 | 0.065792 | 3.13E-13 | 1 | -0.1298 | 0.129803 | -0.1298 | 0.129803 |
| Rainfall (mm) | 0.025665 | 0.076431 | 0.335795 | 0.737408 | -0.12513 | 0.176458 | -0.12513 | 0.176458 |
| Evaporation (mm) | -0.09532 | 0.09817 | -0.97097 | 0.332838 | -0.289 | 0.098364 | -0.289 | 0.098364 |
| Sunshine (hours) | 0.066979 | 0.124297 | 0.53886 | 0.590635 | -0.17825 | 0.312209 | -0.17825 | 0.312209 |
| Speed of max wind (km/h) | 0.145523 | 0.100439 | 1.448868 | 0.149077 | -0.05264 | 0.343683 | -0.05264 | 0.343683 |
| ave temp | 0 | 0 | 65535 | - | 0 | 0 | 0 | 0 |
| sd temp | 0.250898 | 0.12148 | 2.065355 | - | 0.011226 | 0.49057 | 0.011226 | 0.49057 |
| min temp | 0 | 0 | 65535 | - | 0 | 0 | 0 | 0 |
| max temp | -0.1567 | 0.115265 | -1.35946 | - | -0.38411 | 0.070713 | -0.38411 | 0.070713 |
| ave humidity | 0.213194 | 0.115611 | 1.84406 | 0.066782 | -0.0149 | 0.441289 | -0.0149 | 0.441289 |
| ave wind | -0.03385 | 0.099003 | -0.34194 | 0.732784 | -0.22918 | 0.161473 | -0.22918 | 0.161473 |
| ave cloud | 0.179615 | 0.120345 | 1.492501 | 0.13728 | -0.05782 | 0.417048 | -0.05782 | 0.417048 |
| ave pressure | 0.251463 | 0.095586 | 2.630742 | 0.009242 | 0.062877 | 0.44005 | 0.062877 | 0.44005 |
